# Recurrences of advanced sessile and lateral spreading colorectal adenoma after endoscopic mucosal resection (EMR) thermal ablation versus no adjuvant therapy (RESPECT): a protocol of an international randomized controlled trial

**DOI:** 10.1186/s13063-024-07915-2

**Published:** 2024-02-17

**Authors:** Gijs Kemper, Christian Gerges, Erik J. Schoon, Ramon-Michel Schreuder, Ruud R. W. Schrauwen, Ludger S. M. Epping, Torsten Beyna, Joost P. H. Drenth, Ufuk Gündug, Peter D. Siersema, Erwin J. M. van Geenen, the ENDOCARE study group

**Affiliations:** 1 Department of Gastroenterology and Hepatology, Radboud University Medical Center, Radboud Institute for Health Sciences, 6500 HB Nijmegen, The Netherlands; 2 Department of General Internal Medicine and Gastroenterology, Evangelical Hospital Düsseldorf, Düsseldorf, Germany; 3 Department of Gastroenterology and Hepatology, Catharina Hospital Eindhoven, Eindhoven, The Netherlands; 4 Department of Gastroenterology and Hepatology, Bernhoven, Uden, The Netherlands; 5 Department of Gastroenterology and Hepatology, Maasziekenhuis Pantein, Boxmeer, The Netherlands; 6 Department of Internal Medicine and Gastroenterology, Katholisches Karl Leisner Klinikum - St.-Antonius-Hospital Kleve, Kleve, Germany; 7 Department of Gastroenterology and Hepatology, Erasmus MC, Rotterdam, The Netherlands

**Keywords:** Colonic polyps, Endoscopic mucosal resection, Local neoplasm recurrence, Thermal ablation, Randomized controlled trial

## Abstract

**Background:**

Nowadays, large benign lateral spreading lesions (LSLs) and sessile polyps in the colorectum are mostly resected by endoscopic mucosal resection (EMR). A major drawback of EMR is the polyp recurrence rate of up to 20%. Snare tip soft coagulation (STSC) is considered an effective technique to reduce recurrence rates. However, clinical trials on STSC have mainly been conducted in expert referral centers. In these studies, polyp recurrence was assessed optically, and additional adjunctive techniques were excluded. In the current trial, we will evaluate the efficacy and safety of STSC in daily practice, by allowing adjunctive techniques during EMR and the use of both optical and histological polyp recurrence to assess recurrences during follow-up.

**Methods:**

The RESPECT study is a multicenter, parallel-group, international single blinded randomized controlled superiority trial performed in the Netherlands and Germany. A total of 306 patients undergoing piecemeal EMR for LSLs or sessile colorectal polyps sized 20–60 mm will be randomized during the procedure after endoscopic complete polyp resection to the intervention or control group. Post-EMR defects allocated to the intervention group will be treated with thermal ablation with STSC of the entire resection margin. Primary outcome will be polyp recurrence by optical and histological confirmation at the first surveillance colonoscopy after 6 months. Secondary outcomes include technical success and complication rates.

**Discussion:**

The RESPECT study will evaluate if STSC is effective in reducing recurrence rates after piecemeal EMR of large colorectal lesions in daily clinical practice performed by expert and non-expert endoscopists. Moreover, endoscopists will be allowed to use adjunctive techniques to remove remaining adenomatous tissue during the procedure. Finally, adenomatous polyp recurrence during follow-up will be defined by histologic identification.

**Trial registration:**

ClinicalTrials.gov NCT05121805. Registered on 16 November 2021. Start recruitment: 17 March 2022. Planned completion of recruitment: 31 April 2025.

## Introduction

### Background and rationale {6a}

Colorectal cancer (CRC) is the third most common cancer worldwide [1]. In most cases, CRC develops from a premalignant polypoid colorectal lesion [2]. Endoscopic detection and subsequent removal of these polyps prevent CRC-related death [3]. The vast majority can be removed endoscopically during routine colonoscopies. Larger (≥ 20 mm) sessile and lateral spreading lesions (LSLs) require more advanced endoscopic resection techniques. Endoscopic mucosal resection (EMR) is considered the standard of care in the West. However, residual or recurrent adenoma after EMR is a recognized limitation with a frequency at first surveillance of up to 20% [4]. Guidelines advise thermal ablation of the resection margins with snare tip soft coagulation (STSC) [5, 6], which has been reported to reduce adenoma recurrence, from 21% in the control group to 5% in the STSC group (*p* < 0.001), with comparable complication rates [7]. This RCT has however some (design) limitations since no adjunctive techniques were allowed during the procedure and all patients were treated in tertiary referral centers by experienced endoscopists. In addition, optical diagnosis was used instead of histological diagnosis during follow-up after EMR (potentially missing 10% of the recurrent polyps [8, 9].

### Objectives {7}

We will conduct a pragmatic multicenter RCT to evaluate the performance of STSC in daily practice in terms of histological polyp recurrence, technical success, and complication rates.

### Trial design {8}

This study is a multicenter patient-blinded randomized controlled superiority trial in which eligible patients will be allocated to the intervention group with STSC or the control group without additional intervention in a 1:1 ratio. The study protocol adheres to the Standard Protocol Items: Recommendations for Interventional Trials (SPIRIT) guideline [10, 11].

## Methods: participants, interventions, and outcomes

### Study setting {9}

The study will be conducted in both academic and non-academic hospitals in Germany and the Netherlands. A list of study sites can be obtained at https://clinicaltrials.gov/ct2/show/NCT05121805.

### Eligibility criteria {10}

Patients aged ≥ 18 years with a colorectal sessile or LSL (Paris classification 0-IIa/b/c, Is) 20–60 mm in size requiring piecemeal EMR (pEMR) are eligible for inclusion. Eligibility is assessed by the performing endoscopist after inspecting the resection site thoroughly. This study allows adjunctive endoscopic techniques, such as cold snare excision, biopsy forceps, and adjunctive ablation in order to achieve a complete resection. Complete resection is defined as the removal of any visible adenomatous tissue. Exclusion criteria are listed in Table 1.

**Table 1:** Exclusion criteria

*En bloc* endoscopic mucosal resection
Previously attempted resection
Endoscopic appearance of invasive malignancy (non-lifting Kato D, Kudo V pit pattern)
Histologically confirmed malignancy
Presence or suspicion of inflammatory bowel disease
Macroscopic incomplete resection
Located at the ileo-cecal valve or the appendiceal orifice
Contra-indication for surveillance colonoscopy
American Society of Anesthesiology (ASA) grades IV or V
Pregnancy

### Who will take informed consent? {26a}

Potential eligible patients will be identified prior to EMR by the local investigator. Informed consent will be obtained by the local investigator or coordinating investigator.

### Additional consent provisions for collection and use of participant data and biological specimens {26b}

Not applicable. No additional participant data will be collected. No biological specimens will be collected.

### Interventions

#### Explanation for the choice of comparators {6b}

In order to assess the efficacy of STSC to prevent recurrence, we compare patients receiving STSC with patients not receiving STSC.

#### Intervention description {11a}

**Fig. 1:**
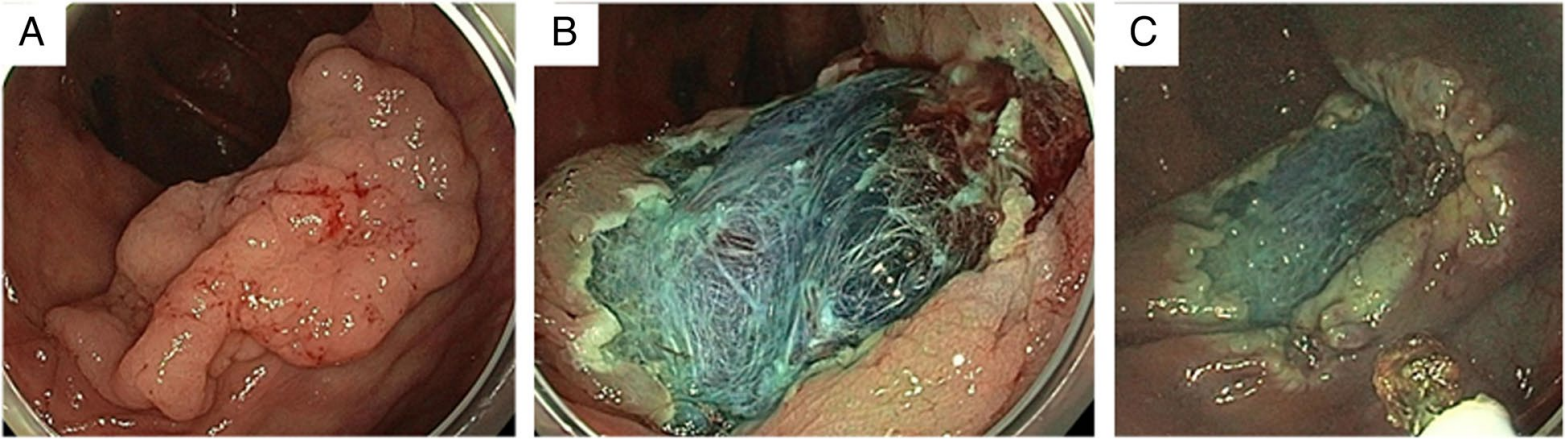
Pre-EMR image of the polyp (**A**), post-EMR image (**B**), and resection site image after snare tip soft coagulation of the peripheral resection margins (**C**). *EMR* endoscopic mucosal resection

Participating endoscopists are instructed to perform STSC by applying 1 to 2 mm of the exposed snare tip directly on the peripheral margins of the EMR site, creating a 2–3-mm rim of ablated tissue around the mucosal defect (Fig. 1). The microprocessor-controlled generator settings will be SOFT COAG mode, 80W, Effect 4 (ERBE Electromedizin, Tübingen, Germany) [7, 12].

#### Criteria for discontinuing or modifying allocated interventions {11b}

Not applicable. The intervention cannot be modified.

#### Strategies to improve adherence to interventions {11c}

Not applicable. Upon informed consent, the intervention is applied one time during the colonoscopy. Patients can withdraw informed consent at any time during the follow-up period but this will not affect the intervention.

#### Relevant concomitant care permitted or prohibited during the trial {11d}

Not applicable. Concomitant care is not prohibited during the trial.

#### Provisions for post-trial care {30}

Not applicable. Post-trial care is not necessary for this trial.

### Outcomes {12}

In accordance with the Dutch polypectomy guideline, the first surveillance colonoscopy (SC1) is performed 6 months after the resection [13]. The primary outcome is the presence of recurrent adenomatous tissue at the resection site at SC1. Potential recurrence will be assessed by careful optical inspection of the scar, followed by (random) biopsies from each scar quadrant in addition to any areas suspicious for recurrence. The local pathologists will assess the biopsies for microscopic recurrence. Polyp recurrence is defined as histological proof of adenomatous neoplastic tissue in the scar biopsies. Secondary outcomes include technical success and complication rates. Patient and procedural-related characteristics including procedure time, the use of adjunctive resection techniques, and the presence of intra-procedural bleedings are collected.

### Participant timeline {13}

The participant timeline is shown in Table 2.

**Table 2:** SPIRIT schedule of enrolment, interventions, and assessments

	Study period			
	Pre-randomization	Primary study period		Follow-up
	− 14 days	pEMR	STSC	+ 180 days
Enrolment				
Eligibility screening	X	X		
Informed consent	X			
Allocation				
Control		X		
Intervention		X	X	
Assessment				
Baseline variables	X			
Procedural variables		X	X	
Primary outcome				X
Secondary outcomes				X

*pEMR* piecemeal endoscopic mucosal resection, *STSC* soft tip snare coagulation

### Sample size {14}

In the colorectum, pEMR has been reported to have a recurrence rate of 20% [4]. Based on the findings of Klein et al. and Kandel et al., we anticipate STSC to be able to reduce the number of recurrences by 60 to 8% [7, 14]. For an 80% power with a 2-sided significance level of 5% and an anticipated drop-out rate of 15%, a total of 306 (2 × 153) patients is required for this study.

### Recruitment {15}

Potential participants will predominately be recruited from national colon screening programs. Identification of eligible patients will be the responsibility of the local principal investigator. Adding additional hospitals to the trial will be considered to ensure sufficient participation enrolment.

### Sequence generation {16a}

Randomization is carried out using a web-based randomization module (Castor EDC, Amsterdam, The Netherlands) and participants are stratified by the center with random block sizes of 2, 4, and 6. Table 2 shows the timing of the enrolment and allocation process.

### Concealment mechanism {16b}

The allocation is revealed once all inclusion and exclusion criteria are met during the colonoscopy. As a result, the allocation sequence is concealed until randomization.

### Implementation {16c}

After endoscopic radical polyp removal, the inclusion and exclusion criteria are re-assessed. When study participation requirements are met, a research assistant not involved in the procedure will randomly assign the patient to the intervention or control group during the colonoscopy. Once the patient is allocated to the intervention group, STSC is performed immediately.

### Who will be blinded {17a}

Patients and pathologists are blinded from the allocated treatment. Due to the study design, endoscopists performing the EMR and the surveillance endoscopy are aware of the allocation. Data analysts will not be blinded.

### Procedure for unblinding if needed {17b}

Not applicable. No need exists for unblinding since only pathologists and patients are blinded.

### Data collection and management

#### Plans for assessment and collection of outcomes {18a}

Data are collected locally on standardized electronic case record forms (eCRF) in Castor EDC by the study coordinators or the local principal investigator. These include patient-related characteristics prior to the pEMR, procedural-related characteristics during the pEMR, and data on the primary and secondary endpoints at the 6-month follow-up time.

### Plans to promote participant retention and complete follow-up {18b}

Participants can leave the study at any time for any reason if they wish to do so. Since the follow-up period is part of standard of care, no additional plans to promote participant retention are made.

### Data management {19}

In order to ensure data quality, all data will be collected and checked by the study coordinator. According to the Dutch Federation of Universities (NFU) standard of risk assessment and monitoring, each participating center will be visited by an independent monitor at pre-defined time points. These monitors will assess the correct handling and storage of study data.

### Confidentiality {27}

Patients will be coded by a numeric randomization code (anonymized) and the principal investigators will be the only ones to have access to this code. The code lists will be stored digitally on the protected hard disc at the local center that included and treated the subject. Its precise location will be written down in the Investigator Site File. The patient data will be recorded in a case record form in CASTOR.

### Plans for collection, laboratory evaluation, and storage of biological specimens for genetic or molecular analysis in this trial/future use {33}

Not applicable. No specimens are collected.

### Statistical methods

#### Statistical methods for primary and secondary outcomes {20a}

The primary outcome will be analyzed according to the intention-to-treat principle with Fisher’s exact test or chi-squared test as appropriate. Recurrences will be reported as recurrence rates (number of cases/total EMRs × 100) for each group. A possible type 1 error will be prevented by including only one lesion per patient. In the unlikely event of a patient having two lesions requiring pEMR and meeting the inclusion/exclusion criteria, only the most distal polyp will be included in the study and the other lesion will be resected during a follow-up colonoscopy. The outcomes of the intention-to-treat analysis will be compared with the per-protocol analysis.

Secondary outcomes include both categorical and continuous variables and will be compared using Student’s *t* test, Wilcoxon rank-sum test, Pearson’s *χ*^2^ test, or Fisher’s exact test as appropriate. For these analyses, a two-tailed *P* < 0.05 will considered to be statistically significant. In case a disbalance exists between potential confounders, multiple regression analysis will be performed for parameters with a *p* ≤ 0.15. Possible risk factors for recurrence will be identified using logistic regression. The Bonferroni correction method will be used to adjust the *p*-value for multiple testing. A cost-effectiveness analysis will be performed in case of superiority of the intervention arm.

### Interim analyses {21b}

We will not perform an interim analysis.

### Methods for additional analyses (e.g., subgroup analyses) {20b}

A sensitivity analysis will compare the histology-proven recurrence rates with the macroscopic recurrence evaluation.

### Methods in analysis to handle protocol non-adherence and any statistical methods to handle missing data {20c}

Missing data will be prevented as much as possible by providing the participating endoscopists with a checklist of the parameters that should be collected during the two colonoscopies. The baseline characteristics are provided by the electronic medical records. The reason for any missing data compromising any outcome will be reported. Depending on the percentage of missing data, parameters with missing data will be imputed or left out of the analysis after consulting a statistical expert.

### Plans to give access to the full protocol, participant-level data, and statistical code {31c}

The full protocol and datasets generated and/or analyzed during the current study are available after publication from the primary investigator on reasonable request.

### Oversight and monitoring

#### Composition of the coordinating center and trial steering committee {5d}

The Radboudumc acts as the coordinating center. The coordinating investigator and the principal investigator together with an expert endoscopist, composing the trial steering committee have monthly meetings to discuss the study progress. The coordinating investigator is responsible for the day-to-day communication with the local principal investigators from the participating centers. Twice a year, members of the Dutch EMR study group are updated on the study’s progress. The coordinating center is responsible for monitoring all participating centers.

### Composition of the data monitoring committee, its role, and reporting structure {21a}

According to the Dutch Federation of Universities (NFU) standard of risk assessment and monitoring, each participating center will be visited by an independent monitor at pre-defined time points. These monitors will assess the adherence to the study protocol and correct handling and storage of study data.

### Adverse event reporting and harms {22}

All adverse events, regardless of a supposed connection to the intervention, will be reported to the study coordinator. In turn, the study coordinator will report adverse events to the Central Committee on Research involving Human Subjects (CCMO) according to Dutch rules and legislation.

### Frequency and plans for auditing trial conduct {23}

Auditing upon invitation of the coordinating hospital board can occur unannounced. The frequency is unknown.

### Plans for communicating important protocol amendments to relevant parties (e.g., trial participants, ethical committees) {25}

Amendments are changes made to the research after a favorable opinion by the accredited medical research ethics committee has been given.

### Dissemination plans {31a}

The trial results will be published open-access. There are no publication restrictions.

## Discussion

The RESPECT study is designed to answer the question of whether STSC after pEMR significantly lowers polyp recurrence rates in daily clinical practice. Although several studies have already investigated STSC performance, some shortcomings have been suggested to impede the generalizability of their findings.

First, only one RCT assessed the effect of STSC on recurrence. This multicenter study included adenomatous polyps ≥ 20 mm removed with complete snare excision, defined as resection of all visible adenomas. As a result, cases in which residual tissue was successfully removed with non-snare adjunctive techniques were excluded [7]. This contrasts with daily clinical practice as adjunctive techniques, such as biopsy forceps and STSC, are regularly used to remove residual tissue. Our study therefore allows the use of adjunctive techniques after pEMR to achieve a macroscopic complete resection of the lesion. Allowing these adjunctive measures, which are a risk factor for local adenomatous tissue recurrence, may reduce STSC performance [15].

Second, the abovementioned RCT was conducted in tertiary centers with highly trained endoscopists and thereby not representing daily practice. The current trial evaluates STSC in both expert and non-expert centers in Germany and the Netherlands.

Third, histological proof of non-recurrence was not always obtained. Biopsies were only taken to confirm areas with suspected recurrences. Recent studies have shown that optical inspection of the post-polypectomy scar may miss residual adenoma in up to 10% [8, 9]. We will therefore assess each scar endoscopically and histologically for recurrent adenoma tissue with random biopsies taken from all four quadrants in addition to any suspicious areas during the surveillance colonoscopy.

STSC has also been evaluated in observational studies. Kandel et al. observed a recurrence rate of 30% in the control group versus 12% in the STSC group after pEMR [14]. In contrast to the RCT, this study allowed cold avulsion for resecting small residual islands of adenomatous tissue. Limitations of this study however include the absence of performing random biopsies for recurrence assessment and its single tertiary center retrospective design. Sidhu et al. conducted a non-comparative prospective cohort study in six tertiary referral centers in Australia, Canada, and Belgium. Thermal ablation of the resection margin resulted in a recurrence rate of 1.4%. Interestingly, incomplete EMR with STSC and the use of adjunctive treatment resulted in a recurrence rate of 27.1% (13/48), showing a lower prophylactic effect of STSC in EMRs combined with adjunctive therapies [16].

Argon plasma coagulation (APC) is an alternative ablative technique and has also been reported to be effective in preventing recurrence [17–19]. Current evidence is however insufficient to identify which technique is superior.

Potential drawbacks of the RESPECT design are the timing of obtaining informed consent. Patients are eligible once a polyp is not yet removed during the initial colonoscopy but scheduled for a second colonoscopy to perform EMR. This may lead to selection bias since smaller or less challenging polyps might be removed in the same session and therefore will not be included in this study.

In conclusion, the RESPECT study evaluates the effect of STSC in daily practice. By allowing adjunctive techniques, assessing recurrence with focused plus random biopsies, and conducting the EMRs in both expert and non-expert centers, we will evaluate the generalizability of STSC in preventing recurrence.

### Trial status

The first patient was randomized on 30 March 2022. To date, 52 patients have been randomized in the participating centers. In the upcoming months, we will start including additional patients in four other centers. Protocol version 7 is being used, and final patient inclusion is expected in April 2025.

## Data Availability

The datasets generated and/or analyzed during the current study are available after publication from the primary investigator (PD) on reasonable request. There are no contractual agreements that limit data access for investigators.

## Abbreviations

CRC: Colorectal carcinoma
LSL: Lateral spreading lesion
EMR: Endoscopic mucosal resection
STSC: Soft tip snare coagulation
RCT: Randomized controlled trial
SPIRIT: Standard Protocol Items: Recommendations for Interventional Trials
pEMR: Piecemeal endoscopic mucosal resection
ASA: American Society of Anesthesiology grade
COAG: Coagulation
SC1: First surveillance colonoscopy
eCRF: Electronic case record form
NFU: Netherlands Federation of University Medical Centres
CCMO: Central Committee on Research involving Human Subjects
APC: Argon plasma coagulation
